# Supplementary material for: Methanogenesis in the presence of oxygenic photosynthetic bacteria may contribute to global methane cycle
Source: Nat Commun. 2024 Jul 6;15:5682. doi: 10.1038/s41467-024-50108-3 (PMC11227571; doi:10.1038/s41467-024-50108-3)
Supplement: Supplementary file 1 — Supplementary Information [file 41467_2024_50108_MOESM1_ESM.pdf]

Supplementary Information for

Methanogenesis in the presence of oxygenic photosynthetic bacteria may contribute to global methane cycle

Jie Ye[1], Minghan Zhuang[1], Mingqiu Hong[1], Dong Zhang[1], Guoping Ren[1], Andong Hu[1], Chaohui Yang[1], Zhen He[2*], Shungui Zhou[1*]

[1]Fujian Provincial Key Laboratory of Soil Environmental Health and Regulation, College of Resources and Environment, Fujian Agriculture and Forestry University, Fuzhou 350002, China

[2]Department of Energy, Environmental and Chemical Engineering, Washington University in St. Louis, St. Louis, MO 63130, USA

* Corresponding author, Email: zhenhe@wustl.edu; sgzhou@fafu.edu.cn

This file includes:

Methods for RNA collection and quantitative RT-PCR quantification

Supplementary Figs. 1 to 19

Supplementary Table 1

Methods for RNA collection and quantitative RT-PCR quantification

The total RNA was extracted and purified using RNeasy Mini Kits (Qiagen Inc., Valencia, CA, USA). Contaminating genomic DNA was digested with DNase, following the manufacturer's instructions (TURBO DNA-free Kit, ThermoFisher Scientific, Waltham, MA, USA). The purified total RNA was then reverse-transcribed into cDNA with random primers using the TransScript First-Strand cDNA Synthesis SuperMix (TransGen Biotech, Beijing, China). Specifically, 300 ng of total RNA was mixed with 0.1 μg random primer, 10 μL of 2× TS Reaction Mix, 1 μL of TransScript RT/RI Enzyme Mix, and nuclease-free water to a final volume of 20 μL. The mixture was incubated at 25 °C for 10 minutes, at 42 °C for 15 minutes, and then heated to 85 °C for 5 seconds to denature the enzyme.

Quantitative PCR was performed using a LightCycler 480 System (Roche, Penzberg, Germany). Reactions were conducted in quadruplicate for each gene tested, in a total volume of 25 μL containing 12.5 μL of iTaq Universal SYBR Green Supermix (Bio-Rad, Hercules, CA, USA), 0.6 mM of gene-specific primers, and 50 ng of cDNA. The real-time PCR was run for 40 cycles with an annealing temperature of 60 °C.

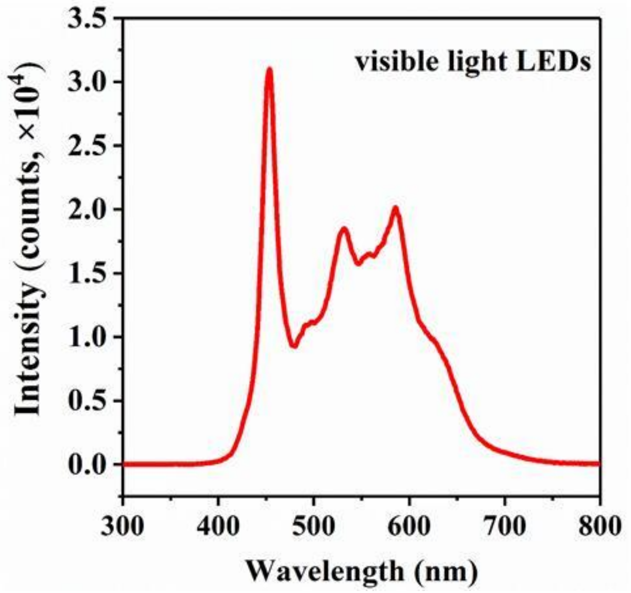

Supplementary Fig. 1 Wavelength spectra of the visible light LEDs. Data are presented as mean values ± SD derived from n = 3 independent experiments. Source data are provided as a Source Data file.

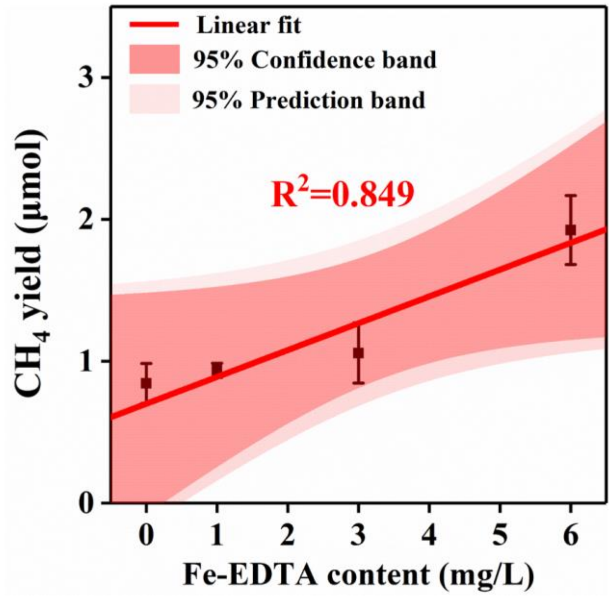

Supplementary Fig. 2 Correlation analysis of CH_4 yield with Fe-EDTA content. Data are presented as mean values ± SD derived from n = 3 independent experiments. Source data are provided as a Source Data file.

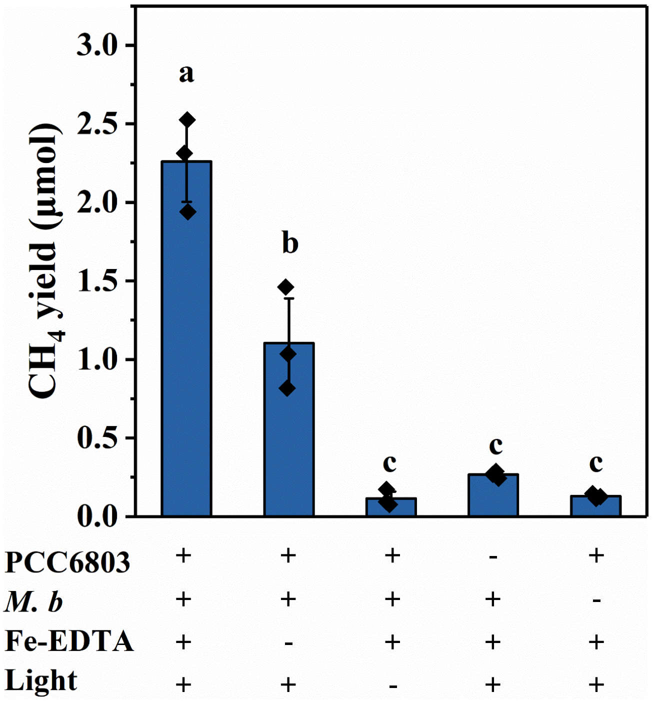

Supplementary Fig. 3 CH_4 yield with PCC6803-*M. b*-Fe-EDTA and deletional controls. "+/-" symbols represent the presence or absence of the relevant component. Data are presented as mean values ± SD derived from n = 3 independent experiments. Statistical analysis was conducted with paired two-tailed *t* tests, and different letters represent statistically significant difference ($P < 0.05$) in different groups. All *P* values are provided in the source data. Source data are provided as a Source Data file.

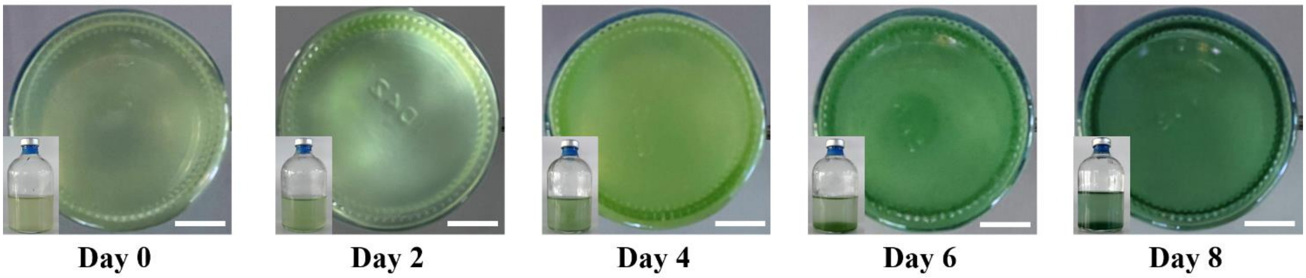

Supplementary Fig. 4 Photographs depicting the evolving biofilm during the coculture. Scale bars: 1 cm.

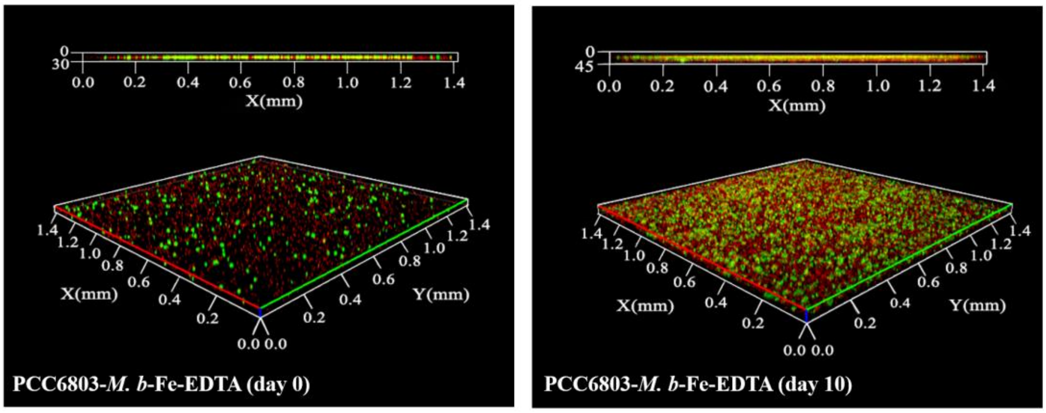

Supplementary Fig. 5 Variation of biofilm thickness with PCC6803-*M. b*-Fe-EDTA after 10 days of syntrophic coculturing; representative of 10 images. The green fluorescence represents live *M. b*, while the red fluorescence represents PCC6803 and dead *M. b*, respectively.

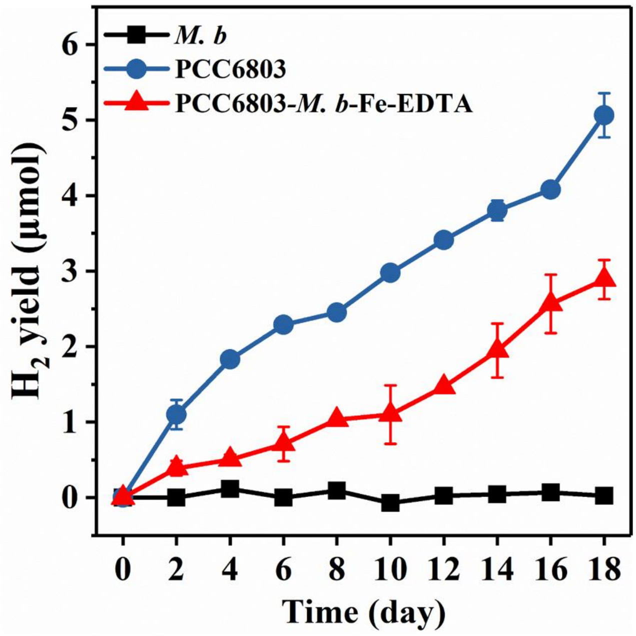

Supplementary Fig. 6 H_2 yield with PCC6803-*M. b*-Fe-EDTA and deletional controls. Data are presented as mean values ± SD derived from n = 3 independent experiments. Source data are provided as a Source Data file.

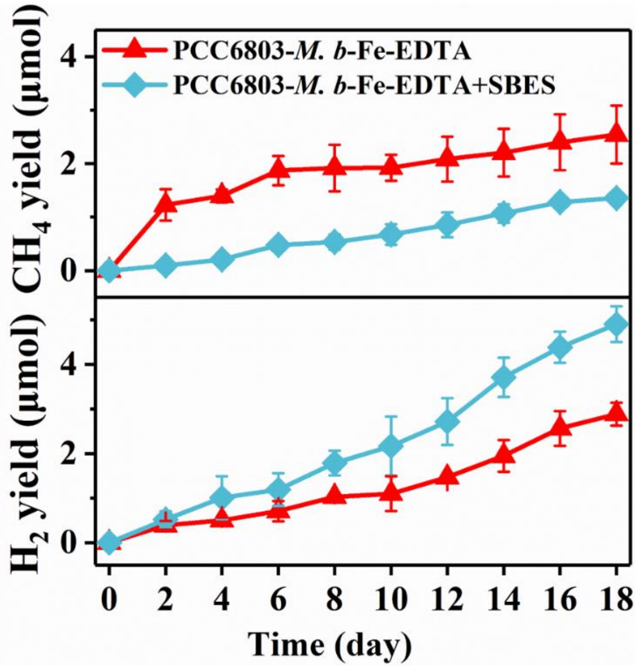

Supplementary Fig. 7 Variation of H_2 and CH_4 yields with PCC6803-*M. b*-Fe-EDTA after SBES addition. Data are presented as mean values ± SD derived from n = 3 independent experiments. Source data are provided as a Source Data file. SBES, sodium 2-bromoethanesulfonate.

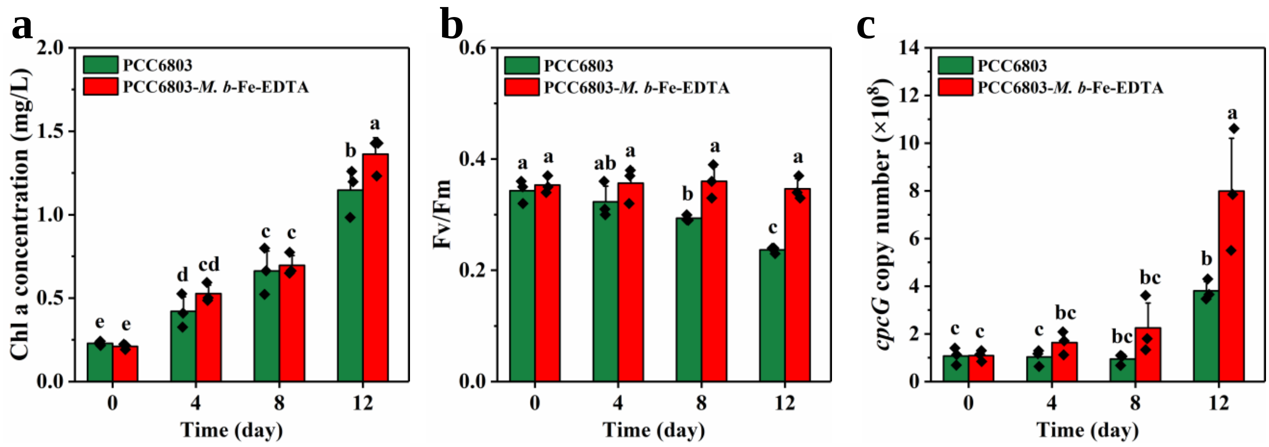

Supplementary Fig. 8 (**a**) Chlorophyll concentration, (**b**) quantum yield of PSII primary photochemical reactions (Fv/Fm), and (**c**) copy number of *cpcG* in bare PCC6803 and PCC6803-*M. b*-Fe-EDTA under a light-dark cycle of 4 h-20 h. Data are presented as mean values ± SD derived from n = 3 independent experiments. Statistical analysis was conducted with paired two-tailed *t* tests, and different letters represent statistically significant difference ($P < 0.05$) in different groups. All *P* values are provided in the source data. Source data are provided as a Source Data file.

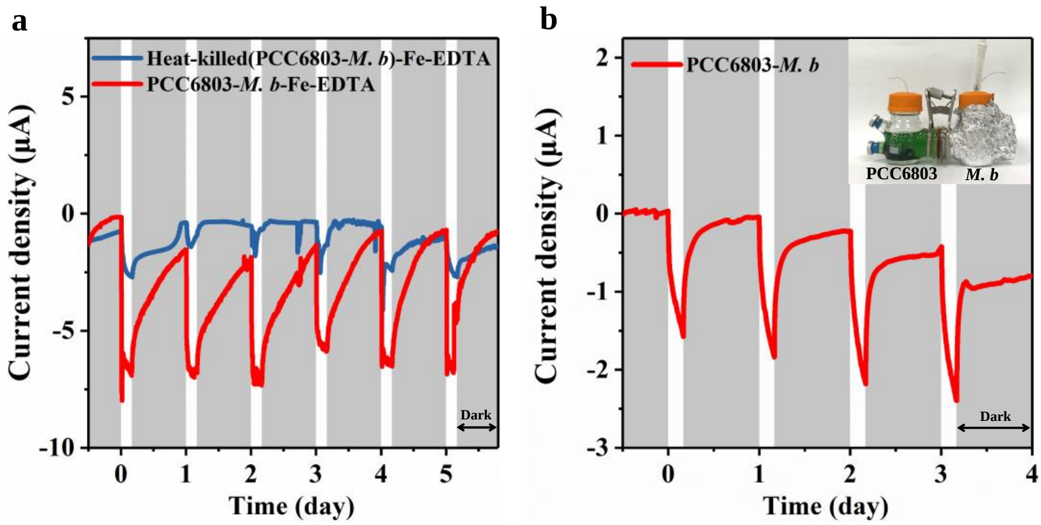

Supplementary Fig. 9 Current densities with PCC6803-*M. b*-Fe-EDTA and deletional controls. (**a**) In single-chamber microbial fuel cell. (**b**) In two-chamber H-cells. External potential bias: -0.5 V; working electrode: carbon cloth with a size of 3 cm × 3 cm; electrolyte: autotrophic medium with/without EDTA-Fe; light on/off cycle: 4 h/20 h; light source: visible light LEDs. Data were recorded every 1 min by the MultiPalmSens4 Potentiostat (PalmSens, The Netherlands). Source data are provided as a Source Data file.

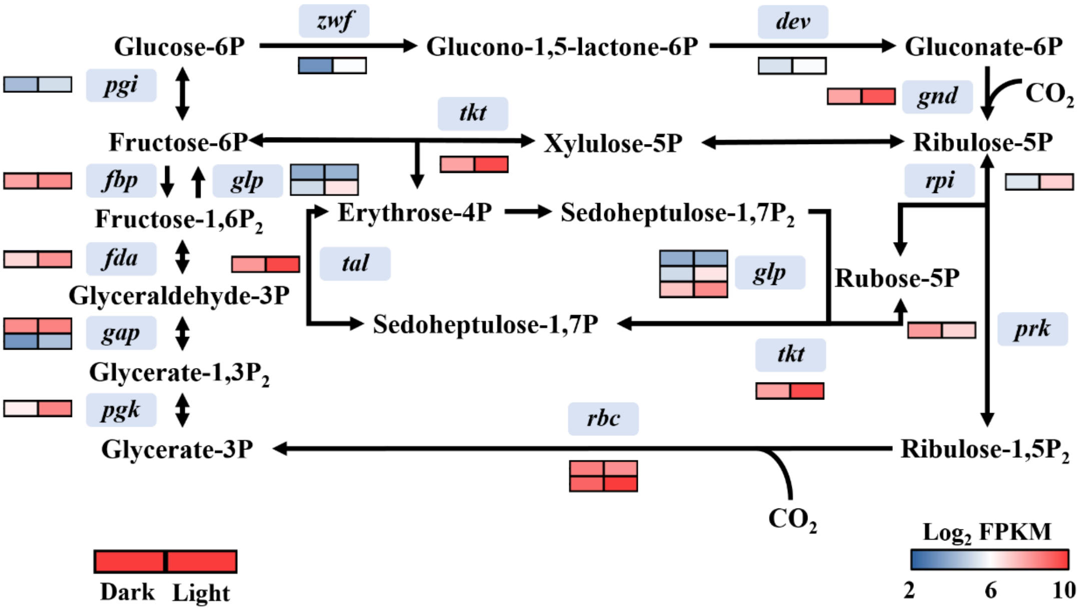

Supplementary Fig. 10 Transcriptomic analyses of key genes encoding carbon fixation. *glk*, glucokinase; *pgi*, glucose-6-phosphate isomerase; *fbp*, fructose 1,6-bisphosphatase; *glp*, GlpX protein; *fda*, fructose-bisphosphate aldolase; *gap*, glyceraldehyde-3-phosphate dehydrogenase; *pgk*, phosphoglycerate kinase; *zwf*, glucose 6-phosphate dehydrogenase; *dev*, glucose-6-P-dehydrogenase; *tkt*, transketolase; *rpi*, ribose 5-phosphate isomerase; *tal*, transaldolase; *prk*, phosphoribulokinase; *rbc*, ribulose-1,5-bisphosphate carboxylase; *gnd*, 6-phosphogluconate dehydrogenase. Source data are provided as a Source Data file.

As shown in **Supplementary Fig. 10**, the key genes for the Calvin cycle, such as RubisCO (*rbc*), phosphoribulokinase (*prk*), and transketolase (*tkt*), had a higher expression during the light period, ensuring efficient CO_2 fixation. Concurrently, the expression of the glycolytic genes was enhanced during the light period, including phosphoglycerate kinase (*pgk*), glyceraldehyde-3-phosphate dehydrogenase (*gap*), fructose-bisphosphate aldolase (*fda*), fructose 1,6-bisphosphatase (*fbp*), and glucose-6-phosphate isomerase (*pgi*). This promoted the glycogen synthesis as a storage molecule in the light period and then use it as a carbon source in the dark period.[1] Furthermore, the genes from the oxidative pentose phosphate pathway (OPPP), such as those encoding glucose-6-P-dehydrogenase (*dev*), transketolase (*tkt*), and glucose 6-phosphate dehydrogenase (*zwf*), were also upregulated in the light period. These genes are intertwined with the activities of the Calvin cycle, resulting in their upregulation during the light phase and downregulation in the dark.[2]

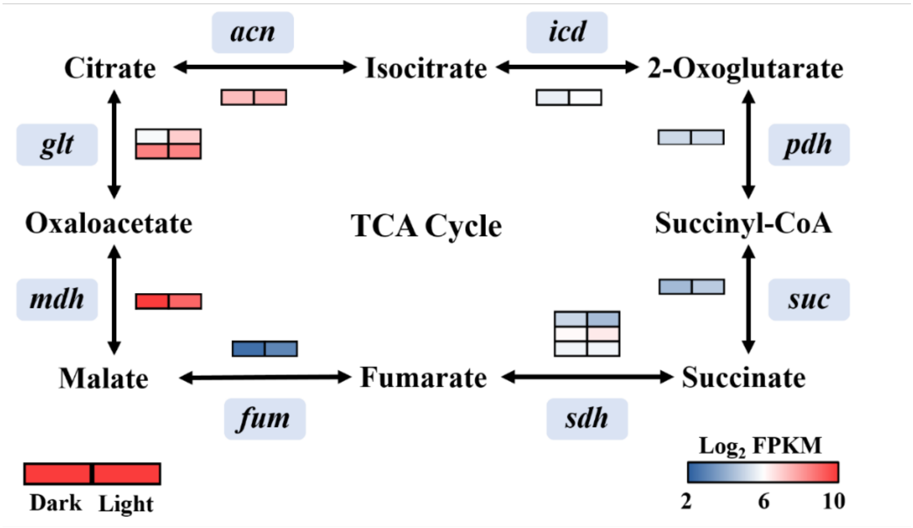

Supplementary Fig. 11 Transcriptomic analyses of key genes encoding TCA cycle. *glt*, citrate synthase; *mdh*, malate dehydrogenase; *fum*, fumarase; *sdh*, succinate dehydrogenase iron-sulfur protein; *suc*, succinyl-CoA synthetase; *pdh*, dihydrolipoamide dehydrogenase; *icd*, isocitrate dehydrogenase; *acn*, aconitate hydratase. Source data are provided as a Source Data file.

PCC6803 possesses a unique tricarboxylic acid (TCA) cycle, with the intracellular citrate levels approximately 1.5–10 times higher than the levels of other TCA cycle metabolite.[S1] *Synechocystis* 6803 is shown to convert citrate to succinate via the TCA cycle during syntrophic methanogenesis,[3] as evidenced by the increased expression of relevant genes (**Supplementary Fig. 11**). For instance, the genes such as *acn* (encoding aconitate hydratase), *icd* (encoding isocitrate dehydrogenase), *pdh* (encoding dihydrolipoamide dehydrogenase) and *suc* (encoding succinyl-CoA synthetase) were upregulated. Notably, *mdh* (encoding malate dehydrogenase) had the highest expression, particularly in the dark, likely contributing to the central role of oxaloacetate as a major "hub metabolites".[S2]

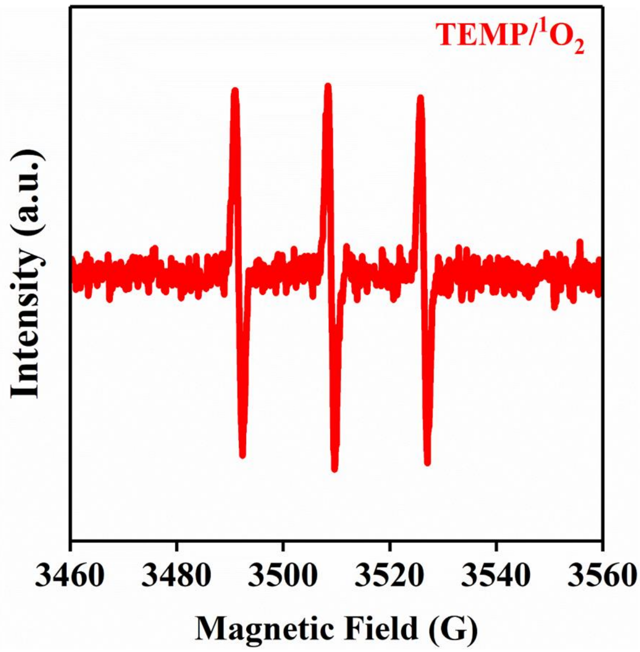

Supplementary Fig. 12 In situ EPR spectrum for $^1O_2$ with TEMP. Source data are provided as a Source Data file.

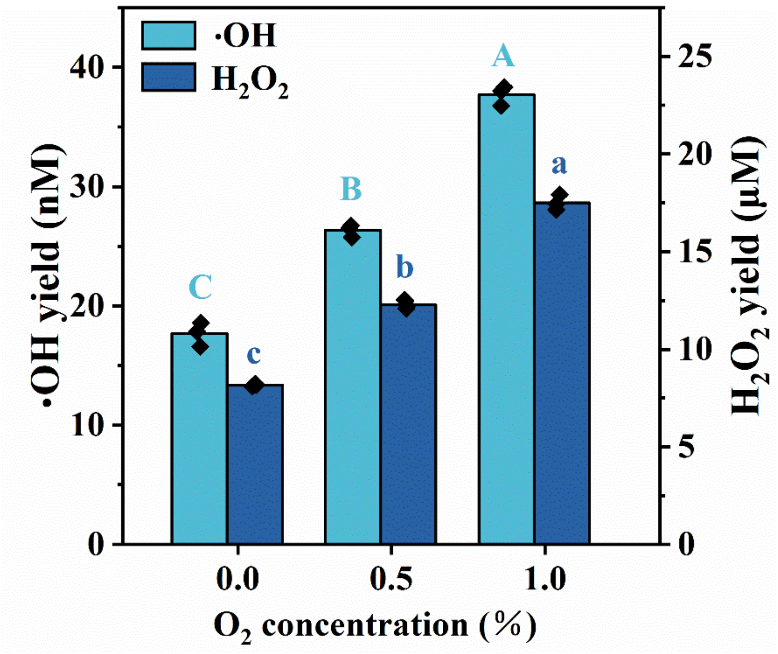

Supplementary Fig. 13 Effects of O_2 concentration on the production of •OH and H_2O_2 by *M. b*. Data are presented as mean values ± SD derived from n = 3 independent experiments. Statistical analysis was conducted with paired two-tailed *t* tests, and different letters represent statistically significant difference ($P < 0.05$) in different groups. All *P* values are provided in the source data. Source data are provided as a Source Data file.

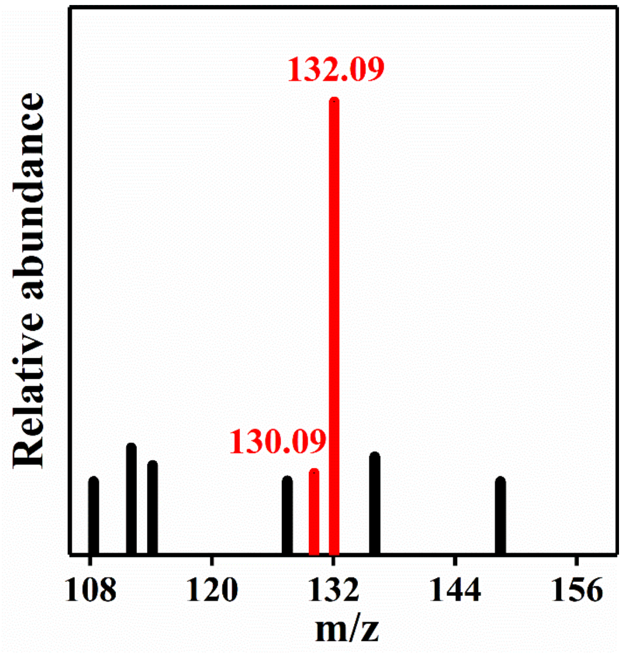

Supplementary Fig. 14 Mass spectra of DMPO-•OH with $^{18}O_2$ as reaction species during ROS production by *M. b*. The mass-to-charge ratios (m/z) of 130.09 and 132.09 represent DMPO-^{16}OH and DMPO-^{18}OH, respectively. Source data are provided as a Source Data file.

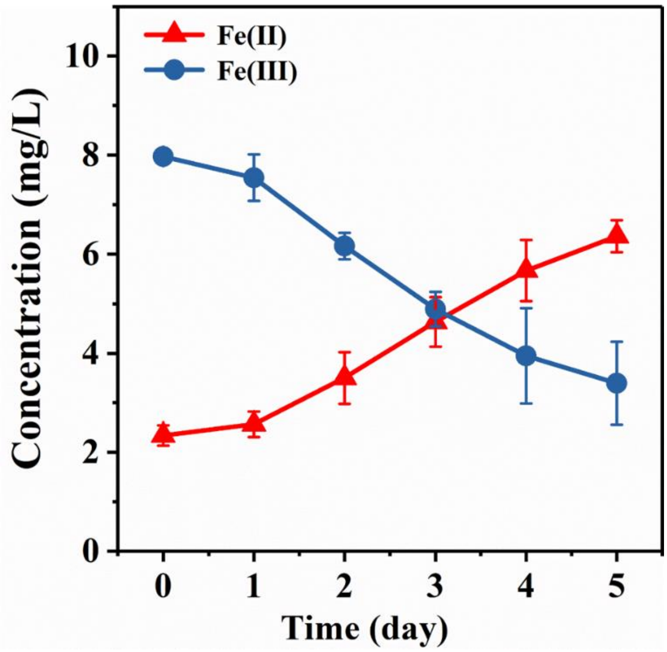

Supplementary Fig. 15 The reduction of Fe-EDTA by PCC6803. Data are presented as mean values ± SD derived from n = 3 independent experiments. Source data are provided as a Source Data file.

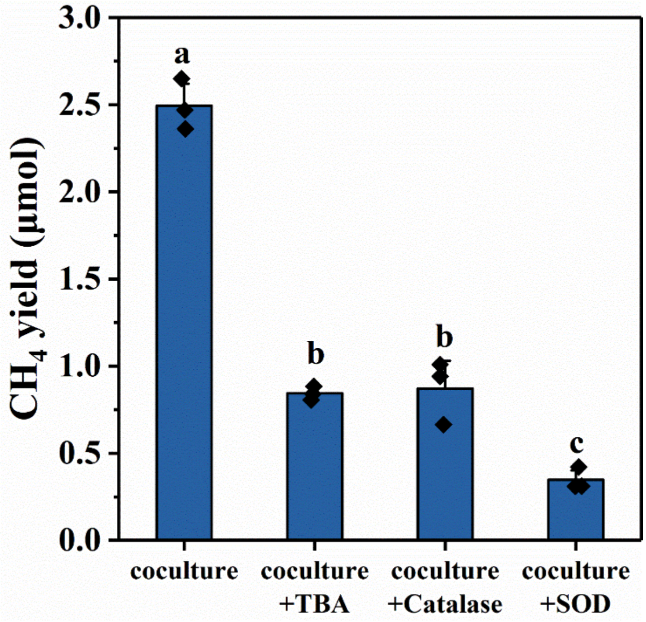

Supplementary Fig. 16 Effects of different ROS quenching reagents on methanogenesis performance after 18 days of syntrophic coculturing. Data are presented as mean values ± SD derived from n = 3 independent experiments. Statistical analysis was conducted with paired two-tailed *t* tests, and different letters represent statistically significant difference ($P < 0.05$) in different groups. All P values are provided in the source data. Source data are provided as a Source Data file. TBA, tert-butyl alcohol; SOD, superoxide dismutase.

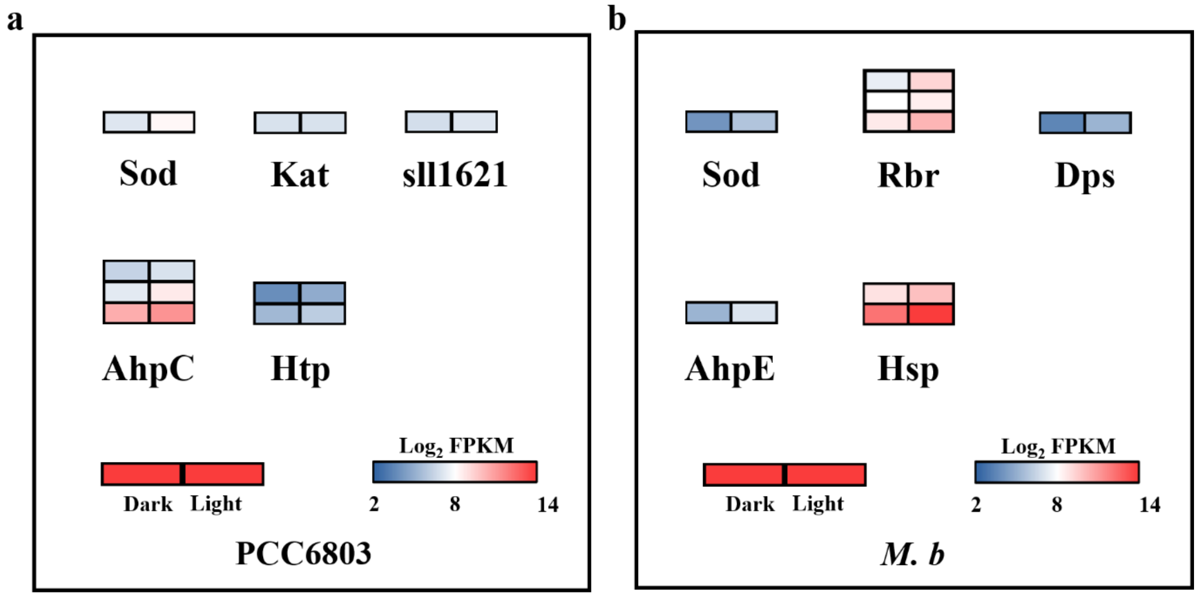

Supplementary Fig. 17 Transcriptomic analyses of key antioxidant stress genes during the syntrophic methanogenesis. (**a**) The transcriptomic levels in PCC6803. (**b**) The transcriptomic levels in *M. b*. Source data are provided as a Source Data file. Sod, superoxide dismutase; Kat, catalase; sll1621, type II peroxiredoxins; AhpC and AhpE, alkylhydroperoxidases; Htp and Hsp, heat shock proteins; Rbr, rubrerythrin; Dps, DNA-binding protein from starved cells.

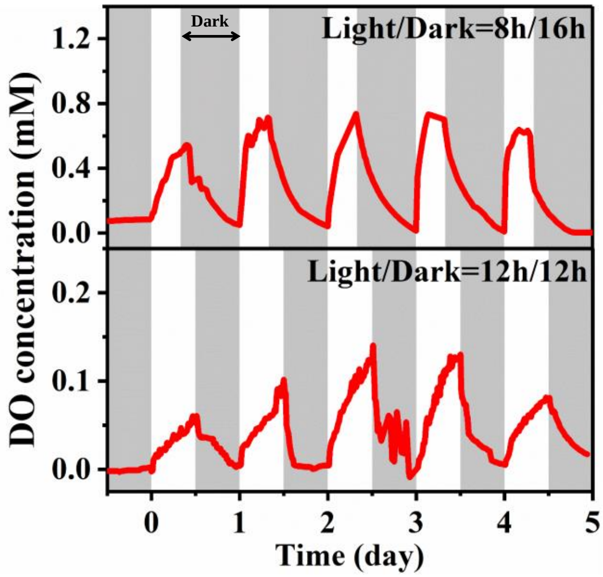

Supplementary Fig. 18 Periodic variation of dissolved oxygen concentration under varied light intensities and illumination times (illumination time of 8 hours with the light intensity of 7 W/m^2, and illumination time of 12 hours with the light intensity of 2 W/m^2). Source data are provided as a Source Data file.

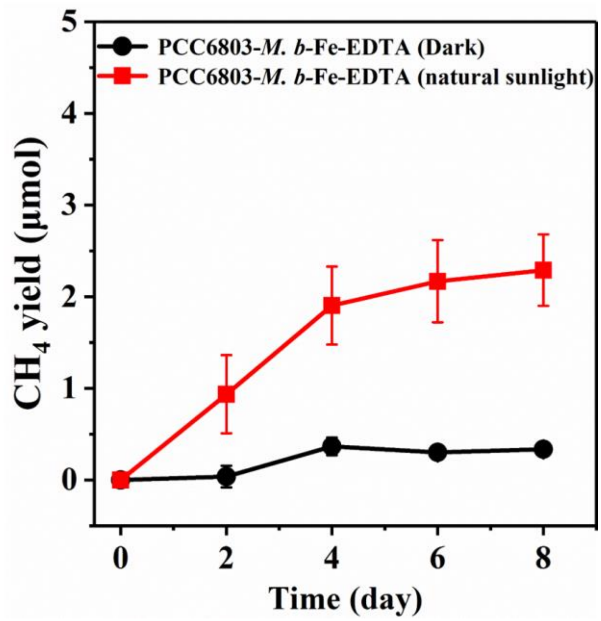

Supplementary Fig. 19 CH_4 yield with PCC6803-*M. b*-Fe-EDTA by natural sunlight irradiation from 08:00 to 20:00 with an average solar heat flux of ~0.5 kW m^{-2} and ambient temperatures ranging between 25 °C and 37 °C. Data are presented as mean values ± SD derived from n = 3 independent experiments. Source data are provided as a Source Data file.

Supplementary Table 1 The composition of contained substrate medium (heterotrophic medium) and uncontained substrate medium (autotrophic medium).

Component	Heterotrophic (g L^{-1})	Autotrophic (g L^{-1})
$MgCl_2 \cdot 6H_2O$	0.4	0.4
$CaCl_2 \cdot 2H_2O$	0.1	0.1
NH_4Cl	0.1	0.1
KH_2PO_4	0.2	0.2
KCl	0.5	0.5
HEPES	7.16	7.16
$NaHCO_3$	2.52	2.52
$Na_2S \cdot 9H_2O$	0.24	–
NaAc	1.394	–
Trace element solution	1 mL	1 mL
ST solution	1 mL	1 mL
Vitamin solution	3 mL VS	3 mL VS

Trace element solution refers to Supplementary Table 1.2. ST solution refers to Supplementary Table 1.3. Vitamin solution refers to Supplementary Table 1.4.

Supplementary Table 1.2 Trace element solution (per liter, the medium containing) that refers to Supplementary Table 1.

Component	Content
HCl (2 M)	50 mL
$FeCl_2 \cdot 4H_2O$	2 g
$ZnCl_2$	0.20 g
$MnCl_2 \cdot 4H_2O$	0.10 g
H_3BO_3	0.18 g
$CoCl_2 \cdot 6H_2O$	0.05 g
$CuCl_2 \cdot 2H_2O$	6 mg
$NiCl_2 \cdot 6H_2O$	72 mg
$Na_2MoO_4 \cdot 2H_2O$	108 mg

Supplementary Table 1.3 ST solution (per liter, the medium containing) that refers to Supplementary Table 1.

Component	Content
NaOH	0.5 g
$Na_2SeO_3 \cdot 5H_2O$	3 mg
$Na_2WO_4 \cdot 2H_2O$	4 mg

Supplementary Table 1.4 Vitamin solution (VS, per liter, the medium containing) that refers to Supplementary Table 1.

Component	Content
4-aminobenzoic acid	0.04 g
DL-a-lipoic acid	0.01 g
Calcium-D(+)-panto-thenate	0.10 g
Pyridoxine-HCl	0.10 g
Folic acid	0.03 g
Nicotinic acid	0.05 g
Riboflavin	0.05 g
Thiamin-HCl·2H_2O	0.01 g
Vitamin B_{12}	0.05 g

Supplementary References

[1] Luan, G., Zhang, S., Wang, M., Lu, X. Progress and perspective on cyanobacterial glycogen metabolism engineering. *Biotechnol. Adv.* **37**(5), 771-786 (2019).

[2] Saha, R. *et al*. Diurnal regulation of cellular processes in the cyanobacterium *Synechocystis* sp. strain PCC 6803: Insights from transcriptomic, fluxomic, and physiological analyses. *mBio* **7**(3), 10-1128 (2016).

[3] Nishii, M., Ito, S., Katayama, N., Osanai, T. Biochemical elucidation of citrate accumulation in *Synechocystis* sp. PCC 6803 via kinetic analysis of aconitase. *Sci. Rep.* **11**(1), 17131 (2021).
