# Supplementary material for: Methanogenesis in the presence of oxygenic photosynthetic bacteria may contribute to global methane cycle
Source: Nat Commun. 2024 Jul 6;15:5682. doi: 10.1038/s41467-024-50108-3 (PMC11227571; doi:10.1038/s41467-024-50108-3)
Supplement: Supplementary file 3 — Reporting Summary [file 41467_2024_50108_MOESM3_ESM.pdf]

nature portfolio

Corresponding author(s): Zhen He, Shungui Zhou

Last updated by author(s): Jun 18, 2024

Reporting Summary

Nature Portfolio wishes to improve the reproducibility of the work that we publish. This form provides structure for consistency and transparency in reporting. For further information on Nature Portfolio policies, see our Editorial Policies and the Editorial Policy Checklist.

Statistics

For all statistical analyses, confirm that the following items are present in the figure legend, table legend, main text, or Methods section.

n/a	Confirmed	
☐	☒	The exact sample size (*n*) for each experimental group/condition, given as a discrete number and unit of measurement
☐	☒	A statement on whether measurements were taken from distinct samples or whether the same sample was measured repeatedly
☐	☒	The statistical test(s) used AND whether they are one- or two-sided<br>*Only common tests should be described solely by name; describe more complex techniques in the Methods section.*
☒	☐	A description of all covariates tested
☒	☐	A description of any assumptions or corrections, such as tests of normality and adjustment for multiple comparisons
☐	☒	A full description of the statistical parameters including central tendency (e.g. means) or other basic estimates (e.g. regression coefficient) AND variation (e.g. standard deviation) or associated estimates of uncertainty (e.g. confidence intervals)
☐	☒	For null hypothesis testing, the test statistic (e.g. *F*, *t*, *r*) with confidence intervals, effect sizes, degrees of freedom and *P* value noted<br>*Give P values as exact values whenever suitable.*
☒	☐	For Bayesian analysis, information on the choice of priors and Markov chain Monte Carlo settings
☒	☐	For hierarchical and complex designs, identification of the appropriate level for tests and full reporting of outcomes
☒	☐	Estimates of effect sizes (e.g. Cohen's *d*, Pearson's *r*), indicating how they were calculated

Our web collection on statistics for biologists contains articles on many of the points above.

Software and code

Policy information about availability of computer code

Data collection	No software was used.
Data analysis	ImageJ software (version 1.51); ZEN software (version 2011); SPSS software (version 19.0); Photon Systems Instruments (AquaPen-C AP-C 100); Keithley Instruments (model 2700).

For manuscripts utilizing custom algorithms or software that are central to the research but not yet described in published literature, software must be made available to editors and reviewers. We strongly encourage code deposition in a community repository (e.g. GitHub). See the Nature Portfolio guidelines for submitting code & software for further information.

Data

Policy information about availability of data

All manuscripts must include a data availability statement. This statement should provide the following information, where applicable:

- Accession codes, unique identifiers, or web links for publicly available datasets
- A description of any restrictions on data availability
- For clinical datasets or third party data, please ensure that the statement adheres to our policy

The data supporting the findings of this study are available within the paper and its supplementary information. The RNA-seq data generated in this study have been deposited in the NCBI Trace Archive database under accession code PRJNA1114667. Source data are provided with this paper.

Research involving human participants, their data, or biological material

Policy information about studies with human participants or human data. See also policy information about sex, gender (identity/presentation), and sexual orientation and race, ethnicity and racism.

Reporting on sex and gender	N/A
Reporting on race, ethnicity, or other socially relevant groupings	N/A
Population characteristics	N/A
Recruitment	N/A
Ethics oversight	N/A

Note that full information on the approval of the study protocol must also be provided in the manuscript.

Field-specific reporting

Please select the one below that is the best fit for your research. If you are not sure, read the appropriate sections before making your selection.

☐ Life sciences ☐ Behavioural & social sciences ☒ Ecological, evolutionary & environmental sciences

For a reference copy of the document with all sections, see nature.com/documents/nr-reporting-summary-flat.pdf

Ecological, evolutionary & environmental sciences study design

All studies must disclose on these points even when the disclosure is negative.

Study description	Our study show unveiled methanogenesis triggered by the interaction between oxygenic photosynthetic bacteria and anaerobic methanogenic archaea. By introducing cyanobacterium Synechocystis PCC6803 and methanogenic archaea Methanosarcina barkeri with the redox cycling of iron, CH4 production was induced in coculture biofilms through both syntrophic methanogenesis (under anoxic conditions in darkness) and abiotic methanogenesis (under oxic conditions in illumination) during the periodic dark-light cycles. We have further demonstrated CH4 production by other model oxygenic photosynthetic bacteria from various phyla, in conjunction with different anaerobic methanogenic archaea exhibiting diverse energy conservation modes, as well as various common Fe-species. These findings have revealed an unexpected link between oxygenic photosynthesis and methanogenesis and would advance our understanding of photosynthetic bacteria's ecological role in the global CH4 cycle. Such light-driven methanogenesis may be widely present in nature.<br>All experiments were replicated three times independently, with consistent results.
Research sample	Cyanobacterium Synechocystis PCC6803 was purchased from the China General Microbiological Culture Collection Center. Methanosarcina barkeri MS (DSM 800) was purchased from the German Collection of Microorganisms and Cell Cultures. PCC6803 is a model oxygenic photosynthetic bacterium that can perform solar energy conversion of water and CO2 to carbohydrates and oxygen, while M. b as a model methanogen was chosen owing to its widespread environmental presence with physiological and metabolic diversity. In addition, oxygenic photosynthetic bacteria Tribonema minus (FACHB-2214, medium BG-11), Euglena sp. (FACHB-1862, Medium HUT) and Chlorella sp. (FACHB-5, medium BG-11) were obtained from the collection of Freshwater Algae Culture Collection at the Institute of Hydrobiology (FACHB), China. Anaerobic methanogenic archaea Methanobacterium bryantii (ATCC33272, DSM medium 1523) and Methanosphaera stadtmaniae (CCAM456, DSM medium 322) were purchase from Biogas Institute of Ministry of Agriculture and Rural Affairs, China. The initial inoculum of Methanococcoides orientis (PRJNA718391) was graciously obtained from the laboratory of Prof. Guangyu Li in Third Institute of Oceanography, Ministry of Natural Resources, China.
Sampling strategy	All experiments were replicated three times independently, with consistent results. In this particular field, reporting data in triplicate is considered a standard practice to enhance the robustness and credibility of the results. Statistical analysis was conducted with paired two-tailed t tests.
Data collection	Minghan Zhuang and Mingqiu Hong conducted the light-driven methanogenesis experiments and measured the concentrations of H2 and CH4 using a Shimadzu Gas Chromatograph (GC-2014, Shimadzu, Japan). Minghan Zhuang and Chaohui Yang performed microscopy with an optical microscope (Nikon Eclipse E200, Japan) and fluorescent in situ hybridization using a UVP HL-2000 HybriLinker hybridization oven. Guoping Ren carried out the quantitative analysis of mcrA and cpcG genes with a Roche LightCycler 480 System (Roche Applied Science, Penzberg, Germany). Transcriptomic data were generated using an Illumina MiSeq, with data analysis conducted by Andong Hu. Minghan Zhuang measured the DO concentration with a UNISENSE OX-NP oxygen needle sensor and photocurrents (I-t) using a data acquisition system (model 2700, Keithley Instruments, Ohio, USA). The compositions of organic substances produced were characterized using Varian INOVA 600-MHz NMR spectroscopy, with data analysis performed by Mingqiu Hong. Reactive species were characterized using a Bruker A300-10/12 EPR spectrometer and Shimadzu UV-2600 UV–Vis spectroscopy. Aqueous Fe(II) concentration was determined via the ferrozine method at a wavelength of 562 nm using the Shimadzu UV-2600 UV-Vis spectroscopy. Guoping Ren measured DMSP by cleaving it into DMS with strong alkali and quantifying DMS by gas chromatography-mass spectrometry (GCMS-QP2020 NX, Shimadzu, Japan).

Timing and spatial scale	This study took place between January 2022 and September 2023. The key experiments are listed as follows: light-driven methanogenesis experiments using a simulated light source were conducted from January 2022 to January 2023, while experiments using actual sunlight were carried out from June 2023 to August 2023. The quantitative analysis of key genes and transcriptomic analysis were performed between February 2023 and June 2023. The composition of organic substances and the kinds of reactive species were measured from April 2023 to July 2023. Electrochemical characterization was conducted in August 2023 and lasted for one month.
Data exclusions	No data were excluded from the analysis.
Reproducibility	Each condition/group underwent at least three biological replicates and similar results were obtained.
Randomization	The site for light-driven methanogenesis experiments with actual sunlight was fully randomized.
Blinding	Every treatment was given unique ID numbers. During parameter measuring, the experimental methods were applied to all samples. During data analyses, the data analysis scripts were applied to all samples simultaneously.

Did the study involve field work? ☐ Yes ☒ No

Reporting for specific materials, systems and methods

We require information from authors about some types of materials, experimental systems and methods used in many studies. Here, indicate whether each material, system or method listed is relevant to your study. If you are not sure if a list item applies to your research, read the appropriate section before selecting a response.

Materials & experimental systems

n/a	Involved in the study
☒	☐ Antibodies
☒	☐ Eukaryotic cell lines
☒	☐ Palaeontology and archaeology
☒	☐ Animals and other organisms
☒	☐ Clinical data
☒	☐ Dual use research of concern
☒	☐ Plants

Methods

n/a	Involved in the study
☒	☐ ChIP-seq
☒	☐ Flow cytometry
☒	☐ MRI-based neuroimaging

Plants

Seed stocks	N/A
Novel plant genotypes	N/A
Authentication	N/A
